# Reactive Nitrogen Species and Male Reproduction: Physiological and Pathological Aspects

**DOI:** 10.3390/ijms231810574

**Published:** 2022-09-12

**Authors:** Sulagna Dutta, Pallav Sengupta, Sanghamitra Das, Petr Slama, Shubhadeep Roychoudhury

**Affiliations:** 1 Department of Oral Biology and Biomedical Sciences, Faculty of Dentistry, MAHSA University, SP2, Bandar Saujana Putra, Jenjarom 42610, Malaysia; 2 School of Medical Sciences, Bharath Institute of Higher Education and Research (BIHER), 173 Agaram Main Rd., Selaiyur, Chennai 600073, India; 3 Physiology Unit, Faculty of Medicine, Bioscience and Nursing, MAHSA University, SP2, Bandar Saujana Putra, Jenjarom 42610, Malaysia; 4 Department of Life Science and Bioinformatics, Assam University, Silchar 788011, India; 5 Laboratory of Animal Immunology and Biotechnology, Department of Animal Morphology, Physiology and Genetics, Faculty of AgriSciences, Mendel University in Brno, Zemedelska 1, 61300 Brno, Czech Republic

**Keywords:** male infertility, oxidative stress, reactive nitrogen species, reactive oxygen species, sperm DNA fragmentation

## Abstract

Reactive nitrogen species (RNS), like reactive oxygen species (ROS), are useful for sustaining reproductive processes such as cell signaling, the regulation of hormonal biosynthesis, sperm capacitation, hyperactivation, and acrosome reaction. However, endogenous levels of RNS beyond physiological limits can impair fertility by disrupting testicular functions, reducing gonadotropin production, and compromising semen quality. Excessive RNS levels cause a variety of abnormalities in germ cells and gametes, particularly in the membranes and deoxyribonucleic acid (DNA), and severely impair the maturation and fertilization processes. Cell fragmentation and developmental blockage, usually at the two-cell stage, are also connected with imbalanced redox status of the embryo during its early developmental stage. Since high RNS levels are closely linked to male infertility and conventional semen analyses are not reliable predictors of the assisted reproductive technology (ART) outcomes for such infertility cases, it is critical to develop novel ways of assessing and treating oxidative and/or nitrosative stress-mediated male infertility. This review aims to explicate the physiological and pathological roles of RNS and their relationship with male reproduction.

## 1. Introduction

Oxidative stress (OS) is induced by a disturbed balance between free radicals and endogenous antioxidant defense. The body is normally protected from OS by enzymatic as well as non-enzymatic antioxidants, which neutralize the excess generated free radical species. Oxidative stress has been linked to a wide range of adverse impacts upon reproductive functions and is involved in the pathogenesis of various diseases such as metabolic syndrome, varicocele, inflammatory diseases, hepatic diseases, and even cancer [1,2,3]. Hepatic diseases such as liver cirrhosis have been seen to be associated with hypogonadism; these patients have gynecomastia, decreased libido, signs of feminization, testicular atrophy, and low testosterone levels, as well as reduced spermatogenesis [4,5]. In a study involving patients diagnosed with Hodgkin’s disease (cancer of part of the immune system, also called the lymphatic system), 70% of patients have been characterized as having baseline seminal abnormalities, including single-parameter defects (24%), combined defects (26%), oligoasthenoteratozoospermia (13%), and azoospermia (8%) [6].

In reproductive biology, reactive oxygen species (ROS) refer to radical or nonradical oxygen derivatives that are vital for germ cell development and function. Since their outer orbit bears an unpaired electron (an exception is hydrogen peroxide (H_2_O_2_), which does not bear an unpaired electron in its outer orbit), both ROS and reactive nitrogen species (RNS) are extremely reactive and interact with a wide range of cellular macromolecules [7,8]. RNS include the nitroxyl ion, peroxynitrite anion, nitrosyl-containing compounds, peroxynitrite, organic hydroperoxide, the hydroxyl radical, nitric oxide, etc. Like ROS, RNS may also cause damage to lipids and proteins via peroxidative and nitration processes. RNS cause lipid peroxidation when membrane phospholipids are brought into contact with them. In this reaction, the free radical oxidizes an unsaturated lipid chain, leading to the formation of a hydroperoxidized lipid and an alkyl radical. This lipoperoxidation results in alterations of the sperm cell membrane structure, affecting its fluidity and damaging its integrity [9]. This is how RNS become harmful to one’s reproductive potential. Such reactions also generate even higher number of free radicals, which, in turn, perpetuates a chain of reactions and results in high levels of OS.

Thus, the male reproductive system has been proven to be negatively affected by RNS, when released in excess amounts [10,11,12], despite the fact that RNS are necessary for a variety of physiological functions in sperm. Nitric oxide (NO) is essential in regulating sperm capacitation and the acrosome reaction—two processes that need to be acquired by sperm in order to achieve fertilization potential [13,14]. There are a substantial number of studies focused on the impact of ROS on male fertility, while this article aims to highlight the role of RNS in the pathogenesis of male infertility. When the concept of nitrosative stress is consolidated, it will be possible to develop potentially effective therapies to treat OS-mediated diseases and will also help produce better outcomes in assisted reproductive technologies (ARTs).

## 2. Mechanism of Formation of RNS

**Figure 1:**
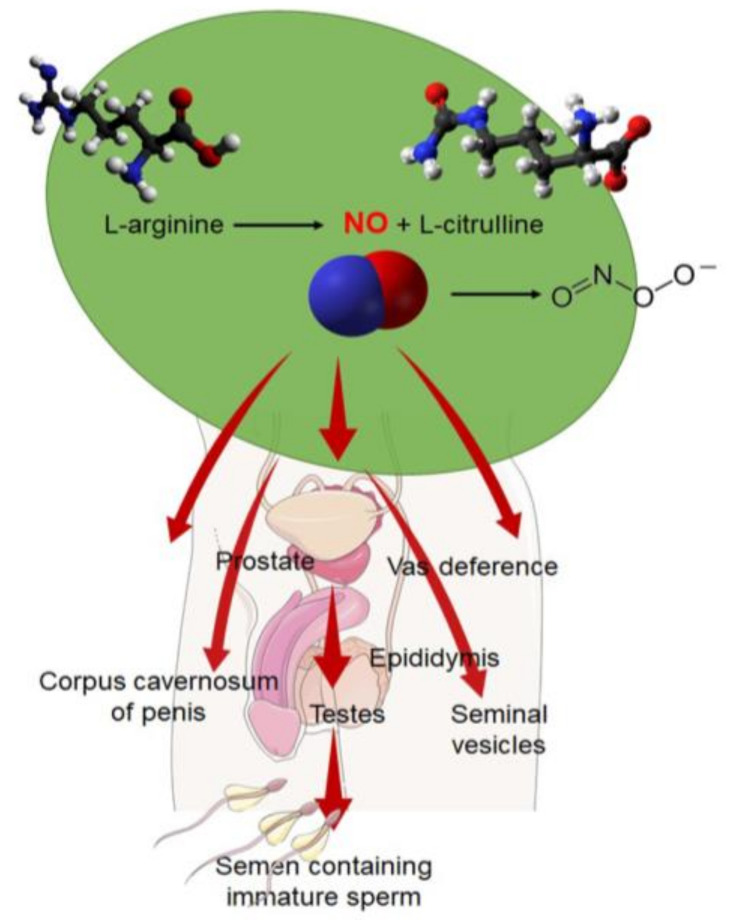
Sources of reactive nitrogen species (RNS). Illustration of the various locations of RNS throughout the male reproductive system. Specifically, these sources can be broken down by structure and various cell types, as shown above. NO—nitic oxide, O_2_—oxygen, ONO_2_—peroxynitrite.

It has been shown that RNS are produced by a variety of sources in the human body, including mesangial and smooth muscle cells as well as hepatocytes and platelets [15]. In some studies, it is also seen that major sources of ROS and RNS are polymorphonuclear leukocytes (PMN) in the semen [16]. RNS are particularly prevalent in the male reproductive tissues, where RNS sources are classified as per the cell types and structure, for example, the male accessory sex glands, epididymis, ducts, testes, penis, and seminal ejaculate (Figure 1). 

L-arginine can be converted into NO and the reaction is mediated by the enzyme nitric oxide synthase (NOS) [17]. NO is produced in the presence of oxygen and an array of cofactors that include flavin mononucleotide (FMN), nicotinamide adenine dinucleotide phosphate (NADPH), calcium, calmodulin, and flavin adenine dinucleotide (FAD), and L-citrulline is also synthesized as a byproduct [15,18]. Three types of NOS are known so far, exerting impacts via protein–protein interactions, and thereby catalyzing the synthesis of NO. The three types are inducible NOS (iNOS), endothelial NOS (eNOS), and neuronal nitric oxide synthase (nNOS). eNOS is the most common type of NOS found in the body. In the reductase domain of each isoform, there is tetrahydrobiopterin (BH4), needed for the generation of NO [15]. Remarkably, the testis-specific nNOS subclass, called TnNOS, has recently been discovered to have an essential role in NO synthesis [19,20]. There is evidence that TnNOS is found exclusively in the Leydig cells of the testicles, indicating that it is involved in the process of steroidogenesis. It has been reported that eNOS and iNOS have a structural association with various tight-junction proteins, including actin, occludin, vimentin, and α-tubulin, indicating that they are vital in modulating the Sertoli cells’ tight junctions, maintaining the blood–testes barrier (BTB) [12] (Table 1). Furthermore, evidence shows that iNOS and eNOS both play integral roles in the apoptosis of spermatogonial progenitors. This concept can be rationalized by the association of eNOS with the degeneration of testicular germ cells, whereas iNOS is associated with germ cell preservation in the seminiferous epithelium. When iNOS is singly considered, it may be considered as a mediator of α-fodrin proteolysis, which promotes the necrosis of germ cells in some cases [12,20].

The expression of iNOS is only induced in the majority of cells following exposure to immune or inflammatory stimuli, for example, via the activated peritoneal macrophages releasing pro-inflammatory mediators such as interleukin (IL)-1 and tumor necrosis factor (TNF)-α [14,19]. This form of NOS is also reportedly triggered via factors released by round spermatids, indicating that testicular germ cells are also part of the regulatory mechanism of the NOS synthesized by the Sertoli and Leydig cells. Throughout the testes, these three isoforms can be found in numerous cell types, including spermatids, spermatozoa, Sertoli cells, Leydig cells, peritubular myoid cells, myofibroblasts, and endothelial cells. Since all variants of NOS, iNOS, eNOS, and nNOS are found in the testes, it may be perceived that NO and RNS are vital for the process of spermatogenesis and other testicular functions [18]. In addition to NOS, an array of chemical compounds, as well as biochemical reactions, have been identified which are involved in the production of NO in the tissues. Research shows that NO synthesis can be mediated by the enzyme glucose-6-phosphate dehydrogenase and via the pentose phosphate pathway (PPP) [22]; more specifically, NADPH, produced by the first reaction of PPP, is needed by NOS to produce NO.

Furthermore, it has been demonstrated that glucose can indirectly stimulate NO production by triggering the PPP and stimulating the reactions to convert L-arginine to L-citrulline [15,25]. Additional research has shown that NO can govern its own function via a feedback inhibitory pathway. In a study, the direct effects of NO were evaluated, using chemically derived S-nitroso-N-acetylpenicillamine (SNAP) and sodium nitroprusside (SNP), on the motility and viability of human spermatozoa. It was seen that a substantial concentration of 0.12 mM for SNAP and a 0.25 mM for SNP had a significant effect on sperm motility, specifically, SNP was found to increase sperm motility [23] (Table 1). Most notably, the interaction of NO with the superoxide anion led to the generation of peroxynitrite, a more damaging oxidant compared to superoxide itself [3,22]. Peroxynitrite and peroxynitrous acid (HONOO) (the breakdown product of peroxynitrite) are severely damaging to sperm and can cause peroxidative damage and the nitrosation of tyrosine molecules, aiding signal transduction [15,26]. Many studies also indicate that peroxynitrite (ONOO-) is harmful at high concentrations for spermatozoa. Typically, these cytotoxic effects are caused by the interaction of these toxins with vital sperm cell components, which essentially include plasma membrane phospholipids, proteins, and peptides, as well as nucleic acids. These adverse effects of ONOO- and HONOO can be mediated directly by initiating uncontrolled oxidative reactions or indirectly via radical-induced processes [11,14,15,25]. Usually, the formation of peroxynitrite occurs only when NO has reached toxic levels and begins to compete with superoxide dismutase for the scavenging of superoxide [11]. Nitrosyl-containing compounds, among others, are important RNS. The reaction between NO and protein thiol groups leads to the formation of S-nitrosyl compounds, which are biologically active and long-lasting. According to the findings, the generation of isoform-dinitrosyl-iron-cysteine could serve as a potential NO mobilization mechanism in the body.

In addition to binding to iron–sulfur clusters and nitrotyrosine generation [11], NO can be transported through additional pathways by binding to the heme-bearing respiratory chain protein. New therapeutic strategies to mitigate the negative consequences of RNS can be created with a greater understanding of their physiological and pathological effects, as well as their processes [27].

## 3. Physiological Significance of RNS

### 3.1. RNS as Cell Signal Transducers

**Figure 2:**
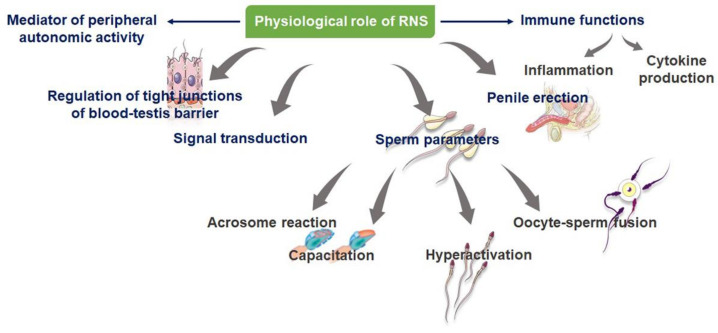
Physiological role of reactive nitrogen species (RNS). The figure shows the essential role RNS play in the human body. At physiological levels, RNS help to stimulate the immune system, maintain normal sperm parameters, and carry out general reproductive functions in the male body.

Reactive nitrogen species, such as NO at the physiological level, are required for a variety of male reproductive functions. Nonetheless, investigations have shown that RNS concentrations, most commonly, the three main isoforms of NOS—eNOS, nNOS, which is linked to intracellular signaling, and iNOS—below one micromolar play a key regulatory role in numerous cellular signaling pathways (Table 1) regardless of the fact that there is insufficient evidence to suggest and support the specific RNS levels that can be deemed pathogenic [27,28]. Nitric oxide interacts directly with soluble guanyl-cyclase to trigger cyclic guanosine monophosphate (cGMP) production, which, again, stimulates cyclic nucleotide-gated channels (as shown in Figure 1), phosphodiesterase, protein kinase G, and other cGMP-regulated enzymes. It is evident that NO at physiological concentrations (lower than one micromolar) may also activate mitogen-activated protein (MAP) kinase pathways [29]. These pathways relay information to effectors, amplify signals, coordinate incoming information from other signaling pathways, and allow for a variety of response patterns [14]. Among their functions are the transmission of information to effectors, as well as the amplification of signals, the coordination of incoming information from other signaling pathways, and the ability to respond in a variety of ways [14] (Figure 2).

Nitric oxide functions as a signaling molecule in sperm function and viability, regulating the molecular events of spermatozoa motility, capacitation, hyperactivation, acrosome fusion with the oocyte, as well as the proper apoptotic process [30,31,32,33,34]. Several NO actions are driven via sGC/cGMP pathways, whereas others are mediated by the tyrosine nitration of key spermatozoa proteins. Tyrosine nitration was revealed to be critical for sperm function, having both negative [35,36] and positive [37,38] impacts on sperm physiology. A negative impact herein refers to the fact that excess tyrosine nitration causes a decrease in sperm motility and the induction of sperm toxicity. A positive impact of tyrosine nitration can be seen in aiding sperm capacitation, which is the most crucial phenomenon in fertilizing ova in the female reproductive tract [37,39]. Furthermore, NO has been demonstrated to have cytoprotective effects in shielding spermatozoa from lipid peroxidation through enhancement of the effects on protein-sulfhydryl groups, rather than causing oxidative membrane damage [23,39].

### 3.2. RNS in the Formation of Blood–Testes Barrier (BTB)

Although NO is a critical mediator of intracellular signaling pathways, it has an essential function in assembling tight junctions for the formation of the BTB, too (Figure 2). The BTB maintains unique chemical composition of the seminal fluid and creates an immune-privileged micro-environment for the developing germ cells. This barrier also serves to prevent toxic substances from passing through the seminiferous tubules of the testicles and into the bloodstream. One of the key regulators of BTB dynamics is NO, which is also important in ensuring proper spermatogenesis and development of the germ cells [14,18,23]. There is also evidence supporting the role of NOS inhibitors in facilitating the compaction of Sertoli cell tight-junction barriers, in order to prevent the passage of spermatocytes and the proper maturation of germ cells from spermatogonia to homozygous haploid spermatozoa [14]. Because of this knowledge about the role of NO in tight-junction dynamics, it is possible to develop targeted male contraceptives that can prevent the maturation of spermatozoa from crossing the BTB, thereby preventing the formation of viable sperm for pregnancy [23,40].

### 3.3. RNS as Male Reproductive Immune Modulators

Immune cells, macrophages, lymphocytes, mast cells, and dendritic cells are generally found in small numbers in the interstitial compartment of the testes [39]. To facilitate spermatogenesis, the testis strikes a balance toward a tolerogenic immunological microenvironment [39]. 

Reactive nitrogen species are one of the main mediators of cytotoxic and pathogenic processes, hormone synthesis, and inflammation facilitation via platelet aggregation prevention and neutrophil adhesion to endothelial cells [24,39]. They also aid the development of normal vascular tone via the NO/guanyl cyclase/cyclic GMP pathways, which is notably important for penile erection, as shown in Figure 2 [3,21,41]. Additionally, NOS acts via the preganglionic sympathetic neurons traversing the spinal cord to the penis, and this implies that NO may play a role in erectile processes at many neuronal strata [39]. Nitric oxide modulates urogenital tract, urinary bladder, urethra, and penile functions, emphasizing its role as a physiological regulator of peripheral-autonomic activities [42]. These suggest that physiological NO levels aid many of the basic functions of the male reproductive system.

When a pathogenic state arises, the immune cells in the testes are typically activated, resulting in functional impairment of the testes [42]. In fact, nearly 30% of testicular biopsies from infertile patients exhibited asymptomatic, localized inflammatory lesions [43].

Infections, chemical noxae, or physical causes (vasectomy and genital trauma) can cause testicular inflammation [39,44,45,46]; however, in most instances, these inflammatory responses are idiopathic. Azoospermic patients’ testicular biopsies usually reveal a lower number of growing spermatogonia and spermatogonial stem cells. Testes with chronic inflammation in infertile patients also had a higher number of cells expressing cytokines such as the TNF α, IL-6, IL-23, and iNOS, among other inflammatory mediators [17,39,40]. The functional relationship between inflammation and testicular pathology is yet to be established. It is widely known that pro-inflammatory chemicals such as cytokines and NO can disrupt cell cycle progression in a variety of somatic cell types, resulting in anti-proliferative and apoptotic consequences [40,47,48]. In a recent study, TNF-α and NO have been shown to contribute to spermatogenesis impairment by hindering spermatogonial growth and function. The findings revealed that TNF-α and NO are able to induce spermatogonial cell cycle arrest in the S-phase [49]. In this experiment, it was seen that TNF-α increased the percentage of GC-1 spermatogonia cells in the S-phase, and concomitantly reduced the percentage of GC-1 cells in the G1-phase of the cell cycle. The fact that the increase in cells in the S-phase is concomitant with a decrease in the G1-phase might be indicative of cell cycle arrest. Oxidative stress generated by TNF-α was reported to decrease cell proliferation and induce cell cycle arrest. Nitric oxide also displayed an effect similar to TNF-α since it arrested the GC-1 cell cycle in the S-phase and induced apoptosis [49].

Thus, NO is a highly reactive species that can modulate the cell cycle, apoptosis, and proliferation, acting as an intercellular as well as intracellular messenger. Low NO concentrations are thought to support anti-apoptotic and cell growth responses, while high NO concentrations induce cell cycle arrest and cellular apoptosis. Testicular interstitial cells generate more NO than the Sertoli cells. The inducible and constitutive isoforms of NOS are expressed by different types of macrophages and T cells. Because iNOS-null mutant mice had a lower rate of spermatocyte death and a higher sperm count and Sertoli cell number, it has been postulated that the NO-NOS system may play a vital role in determining germ cell and Sertoli cell counts, as well as testicular size [50,51].

### 3.4. NO and Sperm Parameters

When it comes to general bodily functions, physiological levels of NO are critical. They are also critical for the performance of numerous sperm functions, including sperm viability, morphology, motility, capacitation, acrosome reaction, and gamete fusion [18,34]. Spermatozoa are subjected to a continuing priming process in the female genital tract, which is known as capacitation. It is characterized by calcium and bicarbonate influx, cholesterol efflux, and a rise in cAMP and pH [10,18,34,40]. It is mediated by NO and involves the phosphorylation of the tyrosine residues of two spermatozoa proteins [33,39]. The acrosome response, which measures the proportion of capacitated sperm, has been shown to increase (*p* = 0.0007) when spermatozoa are incubated with modest doses of sodium nitroprusside, a NO-releasing chemical. Moreover, NO has been shown to affect the activities of lipoxygenase and cyclooxygenase during capacitation, supporting the hypothesis that NO is involved in sperm function [7,52]. A previous study, however, reportedly contradicted this notion and stated that assisted reproduction does not depend on these enzymatic activities [53].

Sperm hyperactivation, capacitation, and acrosome reaction determine proper fertilization [49]. Sperm hyperactivation is determined by sperm flagellar movements in an asymmetric manner, thereby triggering non-linear sperm motility, great amplitude, and a strong propulsive force that allows sperm penetration into the oocyte [33,54] (Figure 2). Several investigations have demonstrated that NO in physiological concentrations aids sperm motility. There are few studies which have failed to reveal any significant NO-mediated alterations in sperm motility. Specifically, it has been precisely stated that NO at concentrations less than one micromolar increase spermatozoa motility [55], while studies have also reported that NO concentrations greater than one micromolar decrease spermatozoa motility [23,39]. Additionally, a study reported that spermatozoa motility remains unaffected by concentrations greater than or equal to one micromolar in either direction [55]. A high level of NO impairs sperm capacitation without impairing sperm viability [56].

The acrosome reaction, which is closely associated with motility, is similarly impacted by the amount of NO present in the body. It is during the process of spermatozoa approaching the ovum that proteolytic enzymes are released by the sperm acrosome. These enzymes possess a sperm binding site in the glycoprotein layer that helps the spermatozoa to properly fuse with ova. Studies have shown that with sodium nitroprusside present, which can release NO [24,57], there is significant increase in the percentage of spermatozoa undergoing the acrosome response [58,59]. In addition to its importance in initiating the acrosome reaction, NO at physiological concentrations is required for zona pellucida binding [60]. Sodium nitroprusside (at concentrations of 10^−7^ to 10^−8^ M) when incubated with spermatozoa were found to promote sperm–oocyte plasma membrane binding. Nitric oxide also plays a role in maintaining proper spermatozoa morphology, which is an important determinant of fertility status and ART outcomes [61].

### 3.5. NO and Sperm Genetics

The large number of polyunsaturated fatty acids (PUFAs), which provide numerous sites for free radical-induced damage, makes the sperm plasma membrane the most vulnerable to OS-induced injury [62]. In an experiment where fresh semen was collected, it was seen that in males, a diet rich in PUFA had a slight effect on their fertilizing capacity. PUFAs probably modify the sperm cell membrane structures and fluidity, and/or make the sperm cell susceptible to peroxidative damage, which, in turn, directly or indirectly affects sperm genetics [63]. Following spermiogenesis, there is minimal cytosolic space with much lower antioxidant capacity. Sperm have weak DNA damage-sensing and repair systems, making them more susceptible to oxidative damage, mainly to their mitochondrial and nuclear DNA [64]. Male-factor infertility, and paternal-factor-mediated risks of congenital malformations and childhood diseases including neuropsychiatric disorders, have all been linked to sperm having high degree of DNA damage and fragmentation [65]. Higher levels of oxidative sperm DNA damage, as well as single/double strand breaks, GC-to-TA transversions, telomere shortening, and disrupted sperm transcriptions, are linked to impaired blastocyst formation, pre- and post-implantation losses, a greater possibility of fetal death, and poor outcomes in normal pregnancies, as well as in ARTs. Paternal sperm DNA/RNA integrity is thus a significant element that must be considered when analyzing the significance of paternal factors in fertilization and pregnancy outcomes, as well as the risk of fetal genetic abnormalities [66].

Due to substantial chromatin condensation, spermatozoa are usually thought to be transcriptionally silent cells. Sperm protamines do not replace all histones, and the mature sperm nucleus still has 15% chromatin domains containing histones that are assembled with DNA in a typical nucleosomal arrangement [67]. Because of the non-random presence of both transcription factors and RNA polymerase, DNA sequences connected to histones may represent active transcription sites in functionally normal sperm cells. Recent findings imply that the male gamete’s ‘residual’ nucleosomal compartment, which has traditionally been disregarded, may be significantly more vital and important than previously considered. Catalase and SOD mRNA levels were found to be considerably greater in sperm samples with higher NO concentrations [31], implying that NO may boost sperm antioxidative defense by increasing catalase and SOD mRNA levels [31]. The data clearly illustrate that changes in the redox status of spermatozoa can cause changes in gene transcription. The increased expression of nucleus-encoded subunits of both complexes I and IV of the mitochondrial electron transport chain, which was seen in spermatozoa samples with greater NO concentrations, add to this notion [68].

## 4. Pathological Effects of RNS

### 4.1. NO and Induction of Apoptosis in Testicular Cells

**Figure 3:**
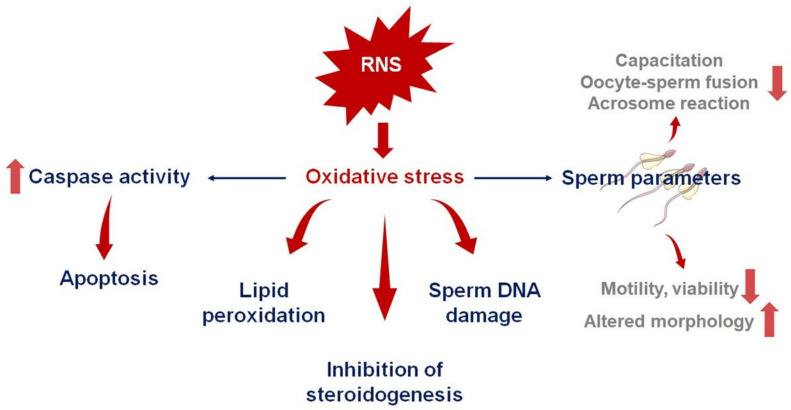
Detrimental role of reactive nitrogen species (RNS). This figure illustrates the harmful role RNS plays in the human body. At toxic levels, RNS can cause a variety of pathological effects on sperm parameters and normal cellular functions.

In addition to germ cell degeneration caused by apoptosis, other issues linked with NO at the pathological levels (10^−4^M) have been reported, as shown in Figure 3 [14]. Apoptosis is a normal physiological response which is essential for the elimination of defective spermatozoa, thereby ensuring normal male reproductive function. In contrast, excessive concentrations of NO cause mitochondrial membrane disruption in spermatozoa, resulting in the release of cytochrome-c and the activation of the caspase pathway, which, in turn, leads to uncontrolled apoptosis. Nitric oxide has been reported to induce apoptosis in the testicular germ cells of mummichog fish (*Fundulus heteroclitus*) via a caspase-dependent pathway, and NO-induced OS synthesized by neuronal NOS synthase is involved in this induction [69].

### 4.2. NO and Steroidogenesis

Levels of NO larger than one micromolar in pathological conditions have also been demonstrated to impede Leydig cell steroidogenesis, according to research (Figure 1). The disruptive action of excess NO on cytochrome P450, which is a cholesterol side-chain cleavage enzyme [14,15,25], can lead to the delayed development of secondary sexual characteristics and the impaired production of sperm, as well as steroidogenesis [14,15,25]. Male infertility and a number of related disorders, such as hypogonadism and metabolic syndrome, can eventually develop from a lack of steroid hormones such as testosterone and dihydroxytestosterone [70].

### 4.3. NO-Mediated Sperm Lipid Peroxidation

Some studies have linked PUFA lipid peroxidation in the sperm plasma membrane to lethal levels of NO (more than one micromolar) [70,71]. In particular, RNS are dangerous to PUFAs since they have hydrogen in their chemical structure, which can easily be extracted. The removal of NO by the body sets off a chain of processes that results in the generation of a free radical, which can then be oxidized further to produce even more free radicals. Lipid peroxidation is the term used to describe this NO-mediated chain reaction [27,72,73,74]. Consequently, the actions of NO are strongly dependent on its level of presence in the male reproductive system.

### 4.4. NO and Impaired Sperm Function

Despite the fact that normal levels of NO aid in the maintenance of normal male reproductive functions, at higher concentrations, NO reportedly has a deleterious impact on numerous sperm parameters (Figure 3). As per the evidence, with sodium nitroprusside present at 10^−4^ M concentrations, the number of spermatozoa attached to the zona pellucida was significantly less compared to that in the control group with no source of NO. A considerable reduction in sperm vitality was reported at the same dosage of sodium nitroprusside; however, a few investigations failed to reveal a statistically significant effect of NO on this sperm parameter [24,75]. Studies also show that hazardous levels of NO impair sperm morphology to a great extent. There are studies which have also shown that NO has no substantial influence on sperm morphology, in a manner similar to that observed in studies on sperm viability [55]. Furthermore, higher seminal concentrations of NO in men with infertility are more likely to result in the inhibition of capacitation in comparison to the seminal plasma NO concentrations in fertile men. Furthermore, greater NO concentrations were found to be associated with a reduced sperm metabolism in infertile men [76]. The body’s natural defense mechanisms must be able to manage the levels of NO in the bloodstream in order to prevent the negative effects of this chemical on sperm activities in general [76].

### 4.5. RNS and Leukocytospermia

At pathological levels, RNS are associated with the occurrence of leukocytospermia (Figure 3). This features the excessive migration and infiltration of leukocytes in the seminal plasma (1 × 10^6^ WBC/mL), which induce OS [24,77,78]. Leukocytospermia is a major causative mechanism leading to male subfertility, and this condition markedly reduces sperm motility by causing sperm agglutination [24]. It is also known that high levels of NO and ONOO- impair sperm motility due to the tyrosine nitration and s-nitrosylation of proteins involved in the generation of energy (enzymes from glycolysis or the Krebs cycle) and those involved in sperm motility (e.g., tubulin) [56]. Moreover, when there are excess active immune cells in the semen, they release cytokines, even higher levels of RNS, and other inflammatory mediators initiating the vicious loop of the inflammation–OS reaction cycle, worsening male fertility parameters. Thus, leukocytospermia leads to pathological levels of RNS, which may trigger OS and inflammatory responses, resulting in related disruptions of semen parameters [24,40,78]. Additionally, leukocytospermia has reportedly been associated with increased ROS production by human spermatozoa.

### 4.6. Varicocele

Considering that RNS regulate a wide range of critical physiological functions at normal levels, excess or insufficient levels of RNS disturb physiological homeostasis and lead to various pathological states. Likewise, higher RNS levels are associated with varicocele, one of the major factors involved in the pathogenesis of male infertility (Figure 3). Varicocele is characterized by swelling and enlargement of the pampiniform vein plexus along the spermatic cord, resulting in decreased circulation in that affected site [79,80,81]. Because of the occlusion of small blood vessels in the scrotum, NO and other RNS are linked to a variety of symptoms of varicocele, including disrupted function of testicular cells such as the germ cells; testicular hypoxia; elevated scrotal temperature; reduced levels of gonadotropins and steroidogenesis; and overall impairment of male reproductive function. While there have been several postulated abnormalities in varicocele, the specific molecular process through which NO functions in this disease remains a mystery [79,80,81].

### 4.7. Erectile Dysfunction (ED)

Reactive nitrogen species may be involved in the mechanism of penile erection, too (Figure 2). Erectile dysfunction (ED) is described as the failure to obtain or sustain penile erections to an extent that can be deemed sufficient to attain fulfilling, complete sexual intercourse [19,82]. Norepinephrine stimulates the soluble guanyl cyclase/cGMP pathway that is activated by NO. This pathway involves the phosphorylation of a variety of proteins, which leads to the relaxation of smooth muscle and filling of blood within the sinusoidal spaces of the penis. The presence of excessive free radicals and an uncontrolled reaction of NO with oxyhemoglobin or superoxide anions results in the synthesis of the damaging peroxynitrite [19,82]. For this reason, there may not be enough NO present to activate the signaling pathways needed for penile erection in ED patients.

### 4.8. Diabetes Mellitus (DM)

Levels of NO that are considered pathological have been linked to diabetes mellitus (DM). This has been specifically related to beta cell death in the pancreas, suggesting that NO may have a role in the hyperinsulinemia associated with type I diabetes. It has been hypothesized that elevated insulin levels in the bloodstream are a result of insulin resistance, and that this increased insulin level may have a role in limiting spermatogenesis and male fertility [26,83]. In a study, DM was associated with very subtle disorders, and affects, either directly or indirectly, various functions of the reproductive system [84]. Semen analysis of diabetic patients (when classified according to their ages and duration of the disease) revealed a decrease in the volume of the ejaculate in the group of younger patients with longer duration of the disease. Additionally, the number of spermatozoa progressively decreased with patients’ age, with a maximal decrease in patients with the longest DM duration. The exact mechanism of the detrimental effects of DM on spermatozoa motility and other related functions has not been elucidated yet [84].

### 4.9. Strategies to Measure RNS 

There are two methods by which RNS can be measured—direct and indirect methods. Direct methods measure RNS, whereas indirect methods measure their oxidized products. Direct assays include chemiluminescence, the nitroblue tetrazolium test (NBT), cytochrome c reduction, flow cytometry, electron spin resonance, and the xylenol orange-based assay [85,86,87,88,89]. Indirect methods include measurement using the myeloperoxidase test, and the measurement of oxidation-reduction potential, lipid peroxidation levels, the levels of chemokines, antioxidants, and antioxidant enzymes by measuring the levels of DNA damage and proteomic alterations [90,91,92,93,94,95,96,97,98,99,100,101,102,103,104,105,106,107,108,109,110,111,112,113,114] (Table 2). 

The total antioxidant capacity (TAC) is a parameter that can be measured by evaluating the reducing ability of various antioxidants present in semen against an oxidative reagent such as hydrogen peroxide and measuring the effect on the substrate. The reaction can be measured using a spectrophotometer or colorimeter, depending on the substrate [115,116]. Most techniques employed to estimate TAC measure the low-molecular-weight, chain-breaking antioxidants and do not include the contribution of antioxidant enzymes (the glutathione group of enzymes, catalase, and superoxide dismutase) and metal binding proteins [96,97] (Table 3).

## 5. Conclusions

Oxidative stress-induced male infertility or subfertility have been among the top research priorities in the field of free radical biology in the last few decades. RNS-mediated OS and subsequent male reproductive disruptions need further detailed interventions to reveal the exact pathogenesis involved. This review has concisely updated the physiological and pathological roles of RNS in male reproduction. Evidently, RNS play vital roles in cell cycle regulation, germ cell development, and normal sperm function, among other processes ensuring normal male reproductive homeostasis. Several studies have demonstrated that a healthy balance between the levels of antioxidant systems and the RNS are essential for mediating normal male reproductive function, including maintaining the BTB, essential signaling pathways, steroidogenesis, spermatogenesis, cellular apoptosis, spermatozoa motility, spermatozoa capacitation and hyperactivation, acrosome reaction, and zona pellucida binding [14,23]. However, when RNS exceed normal levels, they lead to OS, the activation of inflammatory pathways, genomic modifications that cause reduced gonadotropin secretion, sperm DNA fragmentation, and aberrant semen parameters [64]. 

In light of the fact that excess RNS are closely associated with OS-induced male infertility, and that conventional semen analyses fail to reliably diagnose these causes or predict the reproductive outcomes, it is critical that novel ways be developed to both assess and treat OS-mediated male reproductive disorders. It is also evident that the use of antioxidant regimens to treat pathologically high RNS levels in the body may often lead to a reduction in RNS below physiological levels. However, only patients with high levels of DNA damage in their sperm have been shown to experience positive benefits from antioxidant treatment. The use of NO synthesis inhibitors or the addition of NO sources, in addition to conventional treatments for ED and infertility, may prove to be beneficial in the treatment of such conditions caused by the imbalance in endogenous NO levels. These scenarios urge in-depth research for establishing the precise cut-off concentration of RNS that marks the induction of OS, which is considered the underlying cause of 30–80% of male infertility cases. Because high levels of RNS have been linked to decreased fertilization rates, impaired embryonic development, high rates of abortion, and increased morbidity in offspring, a better understanding of the role of RNS may be able to help combat the rising rates of male infertility and declining sperm quality that have been reported in recent years.

## Figures and Tables

**Table 1:** Physiological and pathological effects of reactive nitrogen species (RNS) on male fertility.

Type(s) of RNS	Concentration	Experimental Model	Physiological and Pathological Effects	Reference(s)
TnNOS	-	Human	Aids in the process of steroidogenesis	[19,20]
eNOS	50–100 nM	Human	Aberrant patterns of sperm eNOS expression associated with decreased sperm motility (r = −0.46; *p* < 0.05)	[21,22]
iNOS	>1 mM	Human	Structural association with various tight junction-proteins, including actin, occludin, vimentin, and α-tubulin, vital in modulating the Sertoli cells tight junctions maintaining the BTB	[14]
SNP	(i). 0.25–2.5 mM (ii). 10^−6^ to 10^−4^ M	Human	NO induced decreased in sperm motility (*p* < 0.01) and viability (*p* < 0.05). Reduction of sperm motility in a dose- and time-dependent manner by SNP. Sperm progressive motility, and concentration of motile cells also reduced by all SNP doses (*p* < 0.005)	[23,24,25]
SNAP	0–1.2 nmol/10^6^ spermatozoa	Human	A positive correlation was seen between the concentrations of NO and the percentage of immotile spermatozoa (*p* < 0.01).	[25]

BTB—Blood-testes barrier, eNOS—Endothelial nitric oxide synthase, iNOS—Inducible nitic oxide synthase, SNAP—S-nitroso-N-acetylpenicillamine, SNP- Sodium nitroprusside, TnNOS -Testis specific neuronal nitric oxide synthase.

**Table 2:** Methods of measuring reactive nitrogen species (RNS).

Method(s)	Type	Principle	Advantage(s)	Reference(s)
Cytochrome C reduction test	Direct	Reduction of ferricytochrome C to ferrocytochrome C is used to detect superoxide formation.	Gold standard for measuring extracellular superoxide anions.	[87]
Electron spin resonance (ESR), electron paramagnetic resonance (EPR)	Direct	(i) Magnetic properties of unpaired electrons in free radicals enable them to absorb electromagnetic radiation on application of external magnetic field; this then generates absorption spectra utilizing the energy of the electron spin state, which is measured using ESR spectrophotometers. (ii) Provide direct detection of the “instantaneous” presence of free radical species in a sample. (iii) Play a major role in the assessment of most of the oxidants characterized by very short half-life (nanoseconds to microseconds) usually by using stabilizing molecules called spin-traps/probes.	(i) Used to measure oxidative stress on proteins and lipids. Simple, and have high sensitivity and specificity. (ii) Detects free radicals and paramagnetic molecules. The magnetic field-based EPR detection enables nondestructive (in vitro) and noninvasive (in vivo) measurements of biological samples. EPR spectroscopy, coupled with the use of paramagnetic probes, is a potential technique for accurate and precise determination of ROS concentrations in a variety of biological samples.	[86,90]
Xylenol orange-based assay	Direct	(i) Uses automated analyzer. (ii) ROS in semen oxidizes ferrous to ferric ion and this forms a colored complex with xylenol orange in an acidic medium, the color intensity of which can be measured spectrophotometrically. (iii) Results are expressed in μmol H O2 2 equiv./L.	It is rapid, easy, stable, inexpensive, reliable, and sensitive.	[104]
ROS measurement via chemiluminescence	Direct	(i) Measures real-time production of ROS. (ii) Uses two probes—luminol and lucigenin. (iii) Luminol measures global ROS levels, both extracellular and intracellular (superoxide anion, hydrogen peroxide, and hydroxyl radical). (iv) Lucigenin is specific for superoxide anion and hydroxyl radical.	Chemiluminescence is a robust, sensitive, and specific method.	[105,106,107,108,109,110,111]
Flow cytometry	Direct	(i) ROS measurement of hydrogen peroxide and superoxide anion via flow cytometry. (ii) Dihydroethidium measures intracellular superoxide anion and dichlorofluoroscein diacetate for intracellular hydrogen peroxide.	Requires very low amounts of spermatozoa, and high-specificity intracellular ROS in spermatozoa.	[102,112,113,114]
Endtz test	Indirect	(i) ROS is mainly generated by leukocytes. (ii) Myeloperoxidase is used to stain polymorphonuclear granulocytes, but does not provide any information regarding ROS generation by spermatozoa.	Indirect indicator of excessive ROS generation by leukocytes in semen.	[91,102,104]
Redox potential GSH/GSSG	Indirect	(i) Balance of reduced glutathione and its oxidized form (GSSG) gives an indication of ROS levels in vivo. (ii) GSH/GSSG levels are measured biochemically or using high-performance liquid chromatography.	Can be used to measure oxidative stress in vitro and in vivo.	[92,93]
Thiobarbituric acid assay (TBARS)	Indirect	(i) Measures lipid peroxidation. (ii) Detects malondialdehyde (MDA-TBA) adduct by colorimetry or fluoroscopy.	Simple but non-specific.	[103,104]
Oxidation-reduction potential	Indirect	(i) Measures the redox balance in a given biological system. (ii) It measures all known and unknown oxidants and antioxidants in a given sample.	Can be measured both in seminal ejaculates and in seminal plasma (both fresh and frozen).	[98,99,100]

**Table 3:** Methods of measuring total antioxidant capacity (TAC).

Technique	Principle	Advantage(s)	Disadvantage(s)	Reference(s)
Thiobarbituric acid assay (TBARS)	MDA-TBA adduct detection using colorimetry or fluoroscopy	Simple but non-specific	Rigorous controls are required	[94,95]
Isoprostane	EIA/liquid chromatography–tandem mass spectrometry	Specificity, stable compound	Labor-intensive and expensive equipment required	[115]
HNE-His Adduct ELISA	ELISA	Rapid, helps in quantification	Chances of cross-reactivity	[117,118,119]

HNE-His: hydroxynonenal histidine, MDA: malondialdehyde, TBA: thiobarbituric acid.
